# “*When they see us, it’s like they have seen the benefits*!”: experiences of study benefits negotiations in community-based studies on the Kenyan Coast

**DOI:** 10.1186/1472-6939-15-90

**Published:** 2014-12-24

**Authors:** Dorcas M Kamuya, Vicki Marsh, Patricia Njuguna, Patrick Munywoki, Michael Parker, Sassy Molyneux

**Affiliations:** The Ethox Centre, Nuffield Department of Population Health, University of Oxford, Oxford, OX3 7LF United Kingdom; KEMRI-Wellcome Trust Research Programme, Kilifi, Kenya

**Keywords:** Benefit sharing, Research ethics, Ethical dilemmas, Fieldworkers, Developing countries, Social science, Humanitarian aid

## Abstract

**Background:**

Benefit sharing in health research has been the focus of international debates for many years, particularly in developing countries. Whilst increasing attention is being given to frameworks that can guide researchers to determine levels of benefits to participants, there is little empirical research from developing countries on the practical application of these frameworks, including in situations of extreme poverty and vulnerability. In addition, the voices of those who often negotiate and face issues related to benefits in practice - frontline researchers and fieldworkers (FWs) - are rarely included in these debates. Against this background, this paper reports on experiences of negotiating research participation and benefits as described by fieldworkers, research participants and researchers in two community based studies.

**Methods:**

The findings reported here are from a broader social science study that explored the nature of interactions between fieldworkers and participants in two community based studies on the Kenyan Coast. Between January and July 2010, data were collected using participant observation, and through group discussions and in-depth interviews with 42 fieldworkers, 4 researchers, and 40 study participants.

**Results:**

Participants highly appreciated the benefits provided by studies, particularly health care benefits. Fieldworkers were seen by participants and other community members as the gatekeepers and conduits of benefits, even though those were not their formal roles. Fieldworkers found it challenging to ignore participant and community requests for more benefits, especially in situations of extreme poverty. However, responding to requests by providing different sorts and levels of benefits over time, as inadvertently happened in one study, raised expectations of further benefits and led to continuous negotiations between fieldworkers and participants.

**Conclusions:**

Fieldworkers play an important intermediary role in research; a role imbued with multiple challenges and ethical dilemmas for which they require appropriate support. Further more specific empirical research is needed to inform the development of guidance for researchers on benefit sharing, and on responding to emergency humanitarian needs for this and other similar settings.

## Background

There are numerous challenges in conducting research in developing countries. An area that has attracted particular attention in recent years is the study benefits that should be offered to participants and their communities, and to the population living in and around the geographical area in which the research is being conducted. It is widely accepted that research should be locally relevant, and that benefits should outweigh risks for the participants and communities involved [1]. However, there is a great deal of debate about what counts as a benefit, and on levels and types of benefits Lairumbi et al. (2011) reviewed 7 international and 9 country-specific (Africa) ethics guidelines with a focus on benefit sharing in resource poor settings [2]. They summarise the main debates on benefit-sharing including in relation to: who is receiving the benefits i.e. participants, communities, international community/society; when the benefits are provided (framed on a continuum of time of during and after the research); and the different forms of benefits and who is responsible for their provision [2]. The authors point out that while guidelines are supposed to provide broad normative directives, there are discrepancies in approaches to benefits across these guidelines with “obvious potential to result in differing practical interpretations of the ethical conduct of global health research” ([2] p6).

A useful categorisation of benefits by King (2000) distinguishes between *direct* benefits to participants (therapeutic benefits from the intervention being studied), *indirect or collateral* benefits to participants (benefits as a result of being a research participant e.g. free health care) and *aspirational benefits* from the results of research [3]. These forms of benefits are not mutually exclusive, with some overlaps between direct and indirect benefits. There is, however, some lack of clarity on whether compensation for inconveniences and costs, and the provision of ancillary care – that is, care which research participants need but which is not necessary “to answer the research question nor avoid or mitigate harm resulting from participation in the research” ([4], p0709) - should be seen as a form of benefit or as researchers’ responsibilities.

The Fair Benefits framework, developed by researchers working in research institutions in developing countries in 2001, recommends a broad view of what should count as benefits, to include (direct and indirect/collateral) benefits to participants and populations during and after research [5]. In this account, the range of potential direct and collateral benefits are considered not only to be clinical, but to also include employment, capacity building, long term collaboration and financial rewards from research results. The framework sets out the nature of relationship between research institutions and the host population as collaborative. This concept of collaborative partnership between researchers and populations hosting research is separately expounded in Emanuel et al.’s 2004 paper [6]. Commentators have criticised the fair benefits model for being unclear about how resulting bargains can satisfy certain substantive conditions of fairness [7] or for being weak on how negotiating parties can be assumed to have equal power [8].

Even with these frameworks, teasing out the form, nature and magnitude of benefits that should be considered for participants and host populations for a given study or set of studies is a complex process due to competing interests of research and priority needs of community [9]. In addition, whether researchers and research institutions have any responsibility beyond the remit of their research is a point of contention. Lavery et al. (2010) present a ‘relief from oppression’ guiding principle to aid researchers working in socio-economically disadvantaged populations [10]. This guiding principle concerns researchers’ responsibilities to address unmet needs related to background inequities by relating researcher responsibilities to the strength of the researcher-community relationship. They further make an argument for a humanitarian response – a moral obligation to respond to situations that exacerbate vulnerability of communities [10]. The relief of oppression guiding principle may be an additional useful framework for researchers working in socio-economically disadvantaged populations where there are background injustices of unmet health needs, poverty, and significant levels of disease. However, there are relatively few empirical studies from developing countries on how well these frameworks might work in practice.

Fieldworkers, who are at the frontline of research conduct, may face these issues most starkly and most frequently. Fieldworker (FW) is a term often used in our setting to refer to community members employed in research activities to assist in accessing and following-up participants, and in giving consent information [11, 12]. Employing FWs from a community can in itself be described as part of the indirect benefits of a study for communities, and can facilitate mutual understanding between research centres and their communities [13–15]. However, employment of FWs in research activities is not without challenges. A growing body of literature indicates that fieldworkers’ interface position and roles can present significant ethical challenges and dilemmas including how to respond when faced with overwhelming unmet health care and other needs in the community. There can be potential for fieldworkers to breach confidentiality, and to exploit participants’ trust to meet recruitment quotas [15, 16]. For research in many developing countries, lack of guidelines on appropriate levels of benefits, and on how to respond to ethical dilemmas that FWs and other frontline research staff face, could leave them in emotionally and materially vulnerable positions [17].

This paper discusses the centrality of benefits in research negotiations and participation, as a key theme that emerged from a broader social science descriptive study that explored the nature of interactions between FWs and participants in community based studies. The social science research was developed around two on-going community-based studies conducted at the KEMRI-Wellcome Trust research Programme (KWTRP).

### Study site: KEMRI-Wellcome Trust Research Programme

The KEMRI-Wellcome Trust Research Programme (KEMRI-WT) is a biomedical research centre established in 1989 in Kilifi on the Kenyan Coast. A range of studies are carried out in the research centre including laboratory-based research studies, clinical, psychology, epidemiology, immunology, entomology, public health, and social and behavioural research (http://www.kemri-wellcome.org/). The community of nearly 260,000 residents living in and around the Kilifi County Hospital form the catchment area for many research activities at the centre [18]. The main activities and offices of KEMRI-WT are physically located in and around the Kilifi County Hospital, with research and treatment activities conducted in tandem. A branch of the research centre is strategically located in Nairobi to feed research into appropriate government policy arms.

In an effort to ensure adequate standards of diagnosis and treatment particularly for the paediatric population - and more recently for the adult population - a collaborative working arrangement with the County level Ministry of Health (MoH) in the county underpins long-term strategic support. KEMRI-WT boosts clinical services and infrastructural development for all patients using the health facilities in which research is conducted, regardless of that patients’ involvement in research. Discussion with MOH staff before the start of studies is aimed at ensuring that clinical services required for studies are provided in a way that is not undermining of the health care system. For example the research centre might provide additional support to the MOH in terms of drugs and health personnel in the health facilities where research is happening in order to avoid generating differences in services between participants and non-participants. These efforts, aimed at minimising potential for undue inducement - an excessive offer too good or large to potentially cloud judgement of risky research [19] - are not widely described by the research Programme to the local communities because of concern that community members may feel obligated to reciprocate by participating in research.

All studies conducted by the programme are approved by the national scientific and ethics review committees in addition to institutional - and where necessary external - scientific and ethics review [20]. A comprehensive community engagement strategy [21] includes programme-wide and study specific programs. These are led and coordinated by a team of community facilitators - the community liaison group. For studies with a community engagement component, a study- specific community engagement advisory team (CAST – Communication Advice for Studies) is constituted. The main roles of the CAST group are to advise and provide support for study community engagement activities, and to discuss communication issues that arise during study conduct. Members of CAST group are drawn from the study team (with the principal investigator as the chairperson), the community liaison group, social science researchers and at least one fieldworker (FW) or FW supervisor from the study team.

In the Programme, discussions on the nature and range of benefits have been guided primarily by international guidelines and long-term experiences of researchers working in the area. Relatively recently, there have been efforts to begin to develop institutional guidelines on research benefits. To this end, there is an on-going study focused on study benefits (the ‘benefits study’). The ‘benefits study’ involves in-depth “deliberative” consultations with a range of stakeholders including community members and fieldworkers, the initial findings are reported elsewhere [22], and further data collection and analysis are on-going. The study reported in this paper is a separate one which explored the challenges that fieldworkers face in their day-to-day fieldwork activities. Negotiating research participation with potential participants, and the centrality of benefits in those negotiations, was an emerging theme. This means that there are issues raised in this paper that could not be explored in detail at the time. However, these issues will be taken up in more in-depth on the on-going ‘benefits study’.

Fieldworkers are the largest group of staff at the research centre, forming nearly a third of the staff (243 FWs out of 772 staff^a^) [12]. Fieldworkers are salaried employees of the research centre. They are often recruited from the community where a study is on-going, often the catchment area of approximately 260,000 people living in the area surrounding the county hospital (the Kilifi Health and Demographic Surveillance System; KHDSS). With the main roles of communicating about studies, undertaking consent processes and following-up participants at their homes, it is inevitable that fieldworkers are often drawn into negotiations about research participation and benefits with potential participants, and with other community members. The way in which study benefits play out in these negotiations is important in understanding how FWs undertake their roles, the type of ethical challenges they face, and how they could be supported.

## Methods

The broader social science qualitative study, in which the theme of research benefit negotiations emerged, aimed to explore nature of interactions between fieldworkers and research participants in community-based studies, the challenges that fieldworkers faced, and if and how these challenges were resolved. The research was developed around two on-going community based studies, details of which are summarised in Table 1. The two case studies were:

**Table 1:** **Key features of the two community based case studies**

***Feature***	***The RSV-study***	***The Malaria-study***
Study question/objective	Define and quantify who acquires infection from whom in relation to transmission of respiratory syncytial virus (RSV) in households.	Evaluate the efficacy of a ‘promising’ malaria candidate vaccine against malaria disease in infants and children, and across diverse malaria transmission settings in Africa; aimed to address key safety and efficacy information required for vaccine licensure.
Study design	Basic science descriptive study.	Double blind (observer blind), randomized, controlled, multi-centre study.
Study period	Oct 2009 – June 2010; participant involvement for 6 months.	2008 – 2013 (later extended to 2015); participant involvement for 34 months.
Number of study sites	One site in KEMRI-WT, Kilifi.	Eleven sites in seven African countries.
Study area in Kilifi	One location, 15 kms from the Kilifi County Hospital (KCH), within KHDSS.	Three administrative divisions, 30 kms from KCH, 5 locations, in Kilifi County.
Composition of study team	A total of 16 team members;	Minimum of 47 staff;
	• 10 FWs, 2 data entry clerks, one each SFW, clinician, coordinator, PI and senior researcher,	• 36 FWs, 3 SFWs, 3 clinicians, 2 Medical officers, and one each of study coordinator, PI and senior researcher;
	• Team also included shared staff (lab technicians, drivers) with other projects.	• Team also included shared staff (data entry clerks, lab technicians, and drivers) with other projects.
Participants	Entire household in a defined geographic locality; household - where members living in the same compound and with a common eating arrangement e.g. share meals made from the same kitchen. HH selected if had an infant born after previous RSV epidemic; and at least one elder sibling.	Children aged 6-12 weeks and 5-17 months at first vaccination; 16,000 children across the 11 sites, a minimum of 6,000 in each of the age category;
		For Kilifi site, allocated total of 900 children, 600 and 300 in the 5-17 months 6-12 weeks group respectively.

-  An observational basic science study involving entire household (n = 50 households) looking at transmission patterns of respiratory syncytial virus (RSV) in the households; referred to as the RSV-study; and
-  A malaria vaccine trial involving 900 children divided into two groups, 6–12 weeks and 5–17 months; referred to as the Malaria-study.

The two case studies were in geographically different localities, with the RSV-study located within the KHDSS and the Malaria-study outside the KHDSS, about 30 km from Kilifi County Hospital. A total of 36 FWs and 6 Senior FWs^b^ were employed in the two case studies. All the FWs came from and resided within the study population, and the majority were male (7/10 and 25/26 in the RSV and Malaria-study respectively). Their main roles included sharing initial information with potential participants and carrying out follow-up activities. FWs in the Malaria-study were also involved in identifying and recruiting participants, since the study was conducted in an area without a Demographic Surveillance System. The fieldworkers in the RSV-study were more actively involved in data collection than those in the Malaria-study, as shown in Table 2.

**Table 2:** **Study procedures and FW roles in the two case studies**

	***The RSV-study***	***The Malaria-study***
Study procedures	Follow-up visits at home every 3-4 days; data from each HH member collected at each visit included:	Randomisation to one of three groups
	• Temperature,	• Experimental malaria vaccine and its booster at 1.5 years
	• Nasopharyngeal flocked swab (NFS),	• Experimental malaria vaccine and a different
	• Respiratory illness signs and symptoms,	• Booster dose of either Meningitis and
	• Respiratory rate taken for all children under 5 years,	• Septicaemia vacci;ne; and
		• Three doses of rabies vaccine plus a different booster doses of Meningitis and septicaemia;
		*Procedures*
	• Oral swab (taken at alternate visits (once-a-week).	• Initial physical examination, medical history, anthropometric tests, temperature.
		• Three vaccine doses each a month apart, and
	A demographic and risk assessment questionnaire administered at beginning and end of the study.	• booster dose at 34 months.
		• 5 scheduled blood samples over 3 years; each 2.5mls.
		• Monitoring of minor and serious adverse events; immediate post-vaccination and over time.
		• 6 consecutive follow-up visits post-vaccination at home. Monitoring of minor and serious adverse events.
		• Referral to nearest health facility for common illnesses, and to KCH for serious illnesses as advised by attending clinician.
Fieldworkers roles	FWs Carried out all the study procedures at the households.	FWs Undertook the following roles:
		• Home follow-up visits to monitor minor and serious adverse events.
		• Where necessary, made referrals to the health facilities.
		• Organised for transport and food for all those attending the health facility
		• On rota at the local healthy facilitate to assist with anthropometric measures, temperature, and keeping of research data records
		• Followed-up research defaulters.

Table 3 shows the direct and indirect benefits to participants, and indirect benefits to the wider community that were provided in the two case studies. While health care was provided to all participants (all household members in RSV-study and participating children in the Malaria-study), the level and distribution of benefits differed in the two studies. For example all illnesses (acute illnesses such as diarrhoea, fever; and the first referral visit for chronic such as HIV) were covered for participants in the Malaria-study, while only acute illnesses were covered in the RSV-study. These differences in benefit levels had been planned and approved in view of different levels of risks and inconveniences, and different designs of the two studies; a phase III clinical trial with immediate therapeutic benefits to participants (Malaria-study), and a basic science study involving the entire household (RSV-study) with no immediate therapeutic benefits to participants. It is worth noting that the RSV-study study team were not sure of the appropriate levels of compensation for inconveniences (to entire households) and for discomfort from taking of nasal swabs. They requested, and were given permission by the ERC, to determine the details of types and levels of compensation in consultation with the community liaison group, researchers at the research centre and with community leaders at the time of implementing the study. Fieldworkers were not expected to negotiate for study benefits with participants during initial consent processes or over the course of the studies, although this was not openly stated by researchers, primarily because there was an assumption that fieldworkers would not do this. When asked about benefits by participants, FWs were guided to explain as is written in the informed consent forms (see Table 4).

Social scientists carrying out this study were independent of the case study research teams, although the PIs of the case studies (PN and PM) contributed to study design and writing of this manuscript. DK had over 8 years of managing and coordinating community engagement activities, and training and providing support to fieldworkers at the research centre, with support from SM and VM. The knowledge and experience gained through being involved in the institution in this way were important in shaping the overall research and being aware of the context of the different perspectives of researchers, fieldworkers, and community members.

The main data collection methods used were participant observation, natural and focus group discussions and individual interviews.

**Table 3:** **Anticipated risks and benefits in the two case studies**

	***The RSV-study***	***The Malaria-study***
Risks	• Mild discomfort during NFS taking.	• Detailed side effects - as is typical of vaccine trials - provided in the study protocol and informed consent,
	• Time inconveniences.	• Include severe (such as convulsions, diarrhoea) and mild events (e.g. pain, swelling at vaccination site).
Benefits	For *participants:*	*For participants*:
	• Free medical care for all common illnesses (such as diarrhoea, fever regardless of cause, acute respiratory illness etc) during study.	• Free health care for all routine illnesses (e.g. pneumonia, diarrhoea, URTI), vaccine related or otherwise, injuries), throughout the study period (about 3 years), and 1^st^ visit of chronic illnesses (such as sickle cell disease, HIV, epilepsy). This includes:
	• Clinical visits to every participating household once a month at home.	• Free referral for specialized treatment where required, all costs at government facilities covered while transport is provided for first visit to non-government facilities;
	• Other benefits/token staggered throughout the study period included two chairs to each household, sweets, educational materials and token^1^ at end of study.	
		• All transport to and from the hospital provided by the study team;
		• Meals provided for participant and accompanying parents/guardian for all clinic visits;
	*Community benefits:*	*Communal benefits:*
		• Boosting of three health facilities where the study is based; renovation of existing buildings, providing equipment; boosting of health staff, provision of essential drugs,
	• Boosting local health services through provision of drugs, additional clinical staff.	
	• Water treatment for all communal water points.	
	• Provision of emergency medical aid during cholera epidemic including drugs, staff, referrals.	

^1^Token given at the end of the study were said to be the study teams appreciation to participants for having persevered until the end of the study. They included educational materials, food items, and clothes to family members.

**Table 4:** **Explanation in informed consent forms (ICFs) about benefits**

Case Study	Explanation in informed consent forms
**RSV-study**	*Aspiration benefit of the research*
	A vaccine is not yet available to prevent RSV infection but is under development. In order to know which group to target for vaccination in future when that vaccine becomes available, we need to know how the virus is spread within the community. We aim to closely monitor respiratory infections within members of the household in order to understand who transmits the RSV infection to young children.
	*Are there any advantages to me/my child if we participate? (immediate benefits to participants)*
	You/your child will benefit from close monitoring for any illness by a nurse during the home visits and will be referred to the health centre for further consultation and treatment where necessary. Medical expenses for outpatient treatment of acute illnesses will be paid for and where applicable transport costs and treatment at the Kilifi District Hospital will be met by KEMRI. These benefits will be applicable during the period of participation in this study only
**Malaria-study**	*Aspirational benefit of the research*
	Malaria is a common and serious disease in young children that results in many deaths. This study is a test of a new vaccine called (name of vaccine*) to see if it can prevent malaria. If the experimental vaccine works, then it might become part of the routine program of immunization in Africa. To date, there is no licensed vaccines to prevent malaria, the experimental malarial vaccine (name) is still being tested because it’s not known how well it will work in preventing malaria.
	Even if your child takes part in the study, you must continue to follow the methods you normally use to prevent malaria in your family. Sleeping under an insecticide treated bed net with no holes in it can help protect against malaria. And if your child becomes ill, you should seek medical care as soon as possible.
	*Are there any benefits to me/my child for taking part? (immediate benefits to participants)*
	If your child takes part in the study, and receives the first vaccination, he/she will receive medical care from the day of the first dose of vaccine until the completion of the study. This care will include treatment of any symptoms caused by the vaccine or injuries related to the study procedures, as well as treatment for any acute illnesses during the study period and free of charge. Treatment of chronic (long-term) illnesses/injuries unrelated to the study or study procedure will not be paid for by the study. If your child is found to have such illnesses/injuries, he/she will be treated under the existing government services/programs. If your child has a medical problem that cannot be treated at the nearest health centre, we will refer him/her to colleagues at an appropriate clinic. If this were to happen, we will arrange transport for the first visit to see the doctor. All transport to and from the hospital will be provided by the study team so it should not cost you any money to let your child take part in the trial. You will not receive any direct payment just for participating in this study

*Name of vaccine cannot be disclosed as permission not granted by the pharmaceutical company to do so.

### Participant observation

Participant observation aimed to provide first-hand information on the context in which FWs worked and the type and nature of interactions between FWs and households. DK carried out participant observation in both case studies for a total of 4 months in the RSV-study and 1 month in the Malaria-study, and attended 12 and 4 study meetings in the RSV-study and the Malaria-study respectively. She visited 19 (out of 47) households in the RSV-study, and 30 (out of 160^c^) participant households in the Malaria-study, and accompanied all the 10 FWs on selected days in the RSV-study and 9/36 FWs in the Malaria-study in their daily work in communities.

### Group discussions (natural and focus) and in-depth interviews

Table 5 summarises the number of interviews held and the type of respondents involved. A total of 11 focus group discussions (FGDs) with 64 respondents, 5 natural group discussions with 16 respondents and 7 in-depth interviews with 4 different respondents were held. Table 6 shows the checklist used to guide discussions with community participants and fieldworkers. Areas relevant to this paper discussed with participants in the two case studies included: how decisions to enrol, join and stay (or withdraw) in research were arrived at; who within the household was consulted about participation and what was considered; and what challenges participants faced while participating in the research, including who received what benefits at the household and how they felt about it. For interviews with study teams - PIs, study coordinators^d^, SFWs and FWs - topics covered included: factors that facilitated recruitment and retention of participants into studies; factors that contributed to low recruitment and withdrawals from the research; challenges that study team members faced in conducting the research; and strategies, if any, they used to address the challenges. Data collection continued until a point of saturation where no new themes emerged. All interviews were tape-recorded, transcribed verbatim, and translated in to English.

Respondent-households in the RSV-study were selected from the 19 households DK had previously visited during her participant observation. In this way, DK was not a total stranger and had some idea of the household dynamics. Within this group, households were purposively selected to reflect diversity based on gender (including female and male-headed households) and household arrangements (extended and nuclear families). Respondent-households in the Malaria-study were purposively selected from the geographical area surrounding each of the three health facilities in which the trial was being conducted based on age-group of child. FGDs were held separately with male and female respondents in the Malaria-study to address sensitivities around gender roles and household decision-making for research^e^. In both case studies, discussions were held with the study team members separately; that is fieldworkers and senior fieldworkers, study coordinators and principal investigators.

Written informed consent was sought from all participants in the language they were most comfortable with. All participants, apart from one household, gave consent and signed consent forms for the research. The one household where consent was not provided, we think, was because one of the two senior women was reluctant to participate. The participant who did not provide consent was not interviewed and data was not collected from this household.

To safeguard participants’ privacy and confidentiality, all individual identifiers were removed, and codes were used instead of names in transcripts and in this manuscript.

**Table 5:** **Summary of interview methods and respondents**

Interview type	Respondents
In-depth interviews (IDI)	6 IDIs with RSV-study researchers, two each with PI, Study coordinator, Senior FW
	1 IDI with one FW in the Malaria-study
Natural group discussions	5 Natural (household) group discussions with 16 adults participating in the RSV-study
Focus group discussions (FGD)	3 FGDs with 10 FWs in the RSV-study (one group interviewed twice)
	3 FGDs with 26 Field workers in the Malaria-study
	4 FGDs with 24 participants (grouped per gender) in the Malaria-study
	1 FGD with 5 SFW in the Malaria-study

Box 1: Explanation in informed consent forms (ICFs) about benefits^2^ Name of vaccine cannot be disclosed as permission not granted by the pharmaceutical company to do so.

**Table 6:** Questionnaire guides used in interviews with participants and fieldworkers in RSV-study

Group discussion with participants in case studies		
*Decision making on study participation*		
1	When did you join the study (RSV-study)? How did you come to know about the study?	
2	Since the study involved the whole family:	
	•	how did you know about the study (who informed you about the study, when and where)
	•	How did you make the decision to participate? (who was consulted in the family before the decision to participate was made, why were they consulted
	•	Who was not consulted and why not? ( *Promp*t for communication within the family, who was involved in making the decision and why; who was not involved and why;
	•	What contributed to your decision to join the study?
3	How long did you participate in the study for (a short while then dropped, continued to end of study etc.);	
	•	What made you participate to the end of the study?
	•	Did any of the HH members change their decisions as the study continued? Why? How did you handle this? (probe: how they communicated about their decision)
	•	Did any of the HH members drop out of the study and why? What did other HH members feel about this decision? What was done about the decision (e.g. accepted by the study team, talked out of decision)
4	What were some of the most difficult/challenges that you faced while participating in the study (prompt for:	
	•	Study related e.g. procedures, fears around nasal swabbing (e.g. why they were afraid of the nasal swabs), any other
	•	Study benefits and who received these, and how they felt about them
	•	HH challenges around continued participation e.g. time involvement, school going children,
	•	Study team related issues e.g. frequency of FW visits
	•	Dealing with non-participating community members.
5	How were these challenges overcome? – by who,	
	•	any consultations with the HH members,
	•	What was the FWs/study team involvement in resolution (prompt for whether FWs took extra steps to ensure the issues/challenges were resolved) etc.
*End of study discussion:*		
6	Now that the study has ended,	
	•	What are your views/opinions about the study?
	•	What are your views about the FWs and the study team (i.e. how did they handle any of the questions and challenges that participants faced).
	•	Did you face any issues with community members who were not participating? What issues, how did you handle them? Why do you think those issues arose?
*Future participation in KEMRI research:*		
7	In future if invited to participate in a KEMRI research, would you participate, why/why not? What would you consider in making that decision?	
8	Any recommendations?	
**Interviews with fieldworkers in the RSV-study**		
*Changes in study since last FGD:*		
1	Since the last time we talked, did any of the study procedures change? Did any of your FW roles change, if yes, why and in what ways; what were the implications of such changes?	
*End of study discussion:*		
2	The HH study ended two weeks ago, what activities are you currently involved in?	
3	The last time we discussed, you were not very sure that the study will progress to end. Now that it has ended:	
	•	How do you feel the study performed? Was it a success? In what ways? Did it fail in some ways?
	•	Overall what are your views about the study?
4	The study overall has been a success in retaining participants to the end;	
	•	What contributed to such success? (Prompt for what changed in the study so it progressed to the end successfully? How study benefits contributed? How relationship with FWs and study team/researchers contributed)
	•	What do you think was your contribution in making this study a success?
	•	Did you have HH that withdrew from the study? How many and how did you handle this?
	•	Did you have HH that wanted to withdraw and did not? How many and what happened till they did not withdraw?
	•	Did you have HH that wanted to re-join the study? How did you handle this?
	•	Overall what worked well in this study?
	•	Overall what did not work so well, how was this addressed (e.g. FW support system etc.)
5	On reflections, what was the most challenging situation you faced in this study? How was it handled? What was your role in resolving it? What are your opinions about the way it was resolved?	
6	Performance chart for each FW was introduced as the study progressed, what are your views about it? Is this something that other teams can use?	
7	Now that the study has come to an end; how do you feel about the end of the study?	
	•	How do you relate with the participants?
	•	What are your perceptions on how the participants feel about the study, and the study team?
	•	How do non-participants feel about those who participated?
8	How was end of the study communicated, a) to you b) to the participants c) to the community; what are your views about this process of communication.	
9	What are your plans for the future?	
10	Any recommendations	

### Data analysis

We used a combined inductive and deductive approach to data analysis. An initial framework for analysis was developed around key areas of interest and objectives of the research. This framework was revised through open coding of the data, and themes and concepts generated from the coded data [23]. Data analysis started as soon as the initial interviews were transcribed and cleaned, and continued throughout the study. All cleaned transcripts were uploaded into Nvivo Version 8.0, the software used to organize and manage the data. We chose the most informative fieldworker FGD transcript for initial open coding, as it would provide the most variable themes and categories [24]. Data under each open code were grouped into descriptive themes, and codes were merged, deleted and created as more transcripts were added [25]. Initial codes were compared with those of an independent researcher and differences resolved through discussion and referring to the transcript. For each theme, charts were made across different respondents to compare perspectives. We selected quotes to show diversity of views and to illuminate the main themes under discussion.

### Ethics approval

This study was part of a wider evaluation of the implementation and impact of the Programme’s community engagement strategy which was approved by the local and national Institutional Review Boards (IRBs); the KEMRI Scientific Steering (SSC) committee and the KEMRI Ethics Review Committee (ERC), SCC protocol 1463.

## Results

### The role of benefits in participants’ decisions to join and stay in studies

All respondents (participants, fieldworkers and researchers) talked of the various forms of study benefits, and the contribution of those benefits to making the research happen. Below we draw on the literature to categorise the benefits provided into direct benefits, indirect/collateral benefits and aspirational benefits.

### Direct and indirect benefits to participants

In both case studies, the most common and immediate reason given for joining the study was the immediate access to high quality health care for participants, alongside describing varied depths of understanding of the research itself. This is unsurprising since both case studies provided health care as part of the direct and indirect study benefits to participants (see above) and KEMRI-WT is often thought of as a hospital.

“…they [researchers] told us that if the child becomes sick ‘we will come, take the child for treatment and if we are defeated we will send the child and the mother to another hospital and the child will be treated and the mother will be provided with food, then they will be transported by a vehicle up to here [hospital]. Oh! These are good things, the luggage is heavy on our head and someone wants to help. We were pleased and … I wished I had ten children I would release (consent) them all to go into the hands of KEMRI”, (P3/male/Malaria/FGD13).

Previous positive experiences of having been attended by KEMRI staff either at the health facilities or at the county hospital were narrated in many of the interviews in both case studies. Valued positive experiences included treatment of critically ill children or other family members by KEMRI doctors, the concern and attention those staff gave patients, and relatives being informed about what was going on even if the patient eventually died. These experiences were described as having positively contributed to consent and high retention rates in both case studies; retention rates of 78% (47/760) households in the RSV-study and 83% (748/900) participants in the Malaria-study^f^.

I decided fast [to join the study] because of the situation of my child. He was ever sick and I was ever going to the hospital but did not seem to recover, so when the CHWs [community health worker] told me to come and try (join) KEMRI …I said its better I join and my child is now well and fine. So I am very grateful… (P2/female/Malaria/FGD14)

That study benefits facilitated retention of participants was especially clear in the RSV-study, a basic science non-therapeutic study involving a particularly unpopular and unfamiliar study procedure, taking a sample of nasopharyngeal mucus using a flocked swab. The study team realised early on in the study that meeting recruitment targets and retaining nearly 50 entire households in the study would be difficult given the discomfort of the procedure. Additional pressure for the study team to meet recruitment numbers was due to the relatively short duration of the RSV epidemic (five months), and the possibility that failure to carry out the study as designed would lead to extending it to the next epidemic season and to increased research costs. A comprehensive community engagement strategy to inform different constituencies about the study - community leaders, the entire population of the study area, household heads, and eligible household members - had been rolled out. This was perceived to have been well received in the community and facilitated participation. In addition, provision of relatively simple items such as sweets to children was highly praised by both FWs and participants for encouraging participation and helping calm children and their mothers during nasal swabbing.

Yes, like I don’t know whether to call them benefits or what but I think those sweets, they used to be seen like something very small but it contributed in a big way to the success of this project or this study. Because the children became calmer… later, I saw that the adults sometimes liked the sweets maybe even more than the children … (FW4/male/RSV/FGD06)

### Indirect benefits to the wider community

KEMRI-WT was often highly praised for providing high quality health care compared with the perceived lacklustre services of the public health facilities in which trial activities were based.

“…probably a child is sick at the dispensary, and there is the KEMRI section and that of the government. So on the KEMRI section the child gets quick treatment and the treatment the child gets is that of high quality…and in our hospitals they do not investigate, you just tell the doctor how you are feeling and they just assume its malaria, and give you drugs…” (P5/male/Malaria/FGD13)

Due to the relative wealth of the research centre compared to the surrounding community, long lists of community needs were presented at community engagement meetings over the course of the study. While researchers’ and FWs’ general response was to re-explain study requirements and agreed benefits, they were sometimes able to assist in an emergency. For example in the RSV-study when a cholera epidemic broke out, researchers provided emergency aid to the community, including treating all communal water wells, setting up a temporary emergency ward at the local health facility, and providing clinical services, diagnosis kits, drugs, and ambulatory services for referrals to the county hospital. In explaining this assistance, one researcher noted:

“…you can’t see cholera affecting the place and you just sit back and yet you can do something about it. And you can’t also say now that only 3 of our participants have cholera we are only treating those ones. Then again, if the dispensary lacks paracetamol which is a very basic drug, and we have [it]; we can afford to get paracetamol for them, then it’s our social responsibility to them (community)” (R2/female/RSV/IDI05).

Although the Malaria-study was in a drier area of the county, with perennial food shortages, negotiations for different sorts of benefits were less often described in interviews, compared to the RSV-study. This could be because KEMRI-WT was new in the area of the Malaria-study, and hence expectations of what KEMRI-WT could offer were not as high. It also could be that informing and providing complete health care for participating children, and food and transport for participants and guardians for all clinic visits right from the outset of the study addressed (see Table 3) the immediate health care needs for this population. In addition, the intensity of interactions between FWs and households in the RSV-study (with between 2–4 hours per visit to each participating household twice a week in the RSV-study) could have generated different sorts of relationships and expectations (more kinship-like) which increased pressure for FWs to respond.

### Aspirational benefits of the research

Though less often discussed, some participants described altruistic reasons for joining the studies, which seemed to refer to the eventual societal benefit should the research be ‘successful’. They saw their participation in research as contributing to better health for future generations.

“…malaria has disturbed so many people in this world, children have been dying at a young age, mothers miscarry or children die during delivery because of malaria. So after realizing that there was research being done for preventing malaria so that it does not affect us again, I was really pleased by that issue; and I said it was better to join so that we make a contribution for the vaccine to be found [discovered]…” (P1/male/Malaria/FGD13)

In the Malaria-study, these altruistic reasons for participation seemed to be in addition to expectations of immediate therapeutic benefit from the experimental vaccine. Thus, positive results of the research were anticipated to benefit both current and future generations, as explained by one FW:

“All children will benefit [if the study succeeds] but those who benefit first are the ones who are in the study, because it’s only when the research is proved to work, that’s when the others who are not in the study will benefit” (P6/Female/Malaria/FGD14).

Such high expectations, however, might also have been parents’ way of reassuring themselves that they made the right choice in enrolling their children in an experimental vaccine trial whose outcome was uncertain.

### Expanding expectations and demand for more assistance from participants

There was some shifting of benefits given out over time in the RSV-study, in an effort to retain participants and in response to participants (and community) needs and concerns. Thus chairs were provided to facilitate the taking of nasal swabs, education materials were given to school going children, and insecticide treated bed-nets (ITNs) were distributed to pregnant mothers and all children aged less than five years in the participating households. Over time, it became apparent that the study teams’ provision of such benefits and compensations was feeding into expectations for more.

So with every other household we received very many expectations yah, even for some families, some would refuse to be swabbed and tell you, ‘I have a cold, I can’t make it to the dispensary, why don’t you bring me the drugs here at home so that you can swab me’. So that was really difficult for us (R2/female/RSV/IDI05).

Requests for additional benefits included requests for items that the Ministry of Health (MOH) should provide. For example, some parents threatened to withdraw from the study if pre-natal services^g^ and male-child circumcision services were not provided in the RSV- and the Malaria-study respectively. It was difficult for fieldworkers and researchers in both case studies to know how to respond to participants’ requests and needs: re-emphasising study information, as described below, sometimes seemed inadequate.

There is another one [parent of a child participant] who withdrew the other day, she said that the moment a participant is recruited or taken by KEMRI we concentrate so much on the (participating) kid than the mother or other family members; so there are some other moments she wishes to be handled or taken care of herself, or given the attention the kid is given, but she never gets (FW2/male/Malaria/FGD08).

During our field visits at the end of the RSV-study, we noticed that many household members requested tokens of appreciation for having persevered with the study and possibly also in response to hints from FWs that additional tokens might be provided.

### Negotiating study benefits: a challenge for FWs and frontline research staff

As already suggested above, despite the value placed on them by participants, the provision of study benefits appeared to present significant challenges for researchers and FWs. The study teams, including FWs, knew that they could not extend benefits without proper guidance from the research centre (through the community engagement advice team, or CAST), and from the national KEMRI ERC committee. Some participants’ requests for benefits such as food, school fees and uniforms, and shelter, went beyond what the study team, and the research programme, felt they could respond to. Feeling frustrated, fieldworkers sometimes responded in personal ways, including through using their own money to buy food for families. While this provided temporary relief for the participants, the unanswered issue for researchers and the research centre was whether there was a need for a more carefully considered and agreed approach to handle such emergencies for individual households.

With regards to extending benefits and emergency support to individuals, a contested area was whether those already participating in research could be unduly influenced through provision of more and different sorts of benefits. Some researchers felt strongly that enrolled participants could not be unduly influenced as they had already weighed up the risks and benefits for participation. They felt that undue inducement arguments were sometimes used to reduce or deny participants benefits:

“…if I were to decide for this study about the benefits to be given, having been at these households, I would have a very long list of cheap things we can give the households and they would appreciate. Although I know it would be like we are inducing them to participate in the study but they are already participating in the study, so I don’t think introducing extra benefits at this time would have much effect on their participation…” (R2/female/RSV/IDI02)

Some argued that given the relative wealth of the institution, the long-term presence of the research centre in this community, and the high poverty levels and unmet health needs among the community, studies should give more benefits to participants and communities.

“…but that issue (of additional benefits for participants) kept coming up, I think, in practically all of our meetings…and in the long run, each one of us who actually got into contact with those participants or within those households, I think we all felt that we did not give enough… This is a big study, they (participants) have made it succeed, it’s them who have made it a success, so we should also be able to give them something tangible” (R2/female/RSV/IDI05).

Other researchers felt that one way to avoid undue inducement was to give additional benefits to whole communities rather than individuals. A dilemma, where emphasis was placed on community-level benefits, is that participants might be unwilling to participate unless they also receive separate forms of benefits.

“…I believe the idea of balancing (risks and benefits) is trying to ensure that people don’t participate in your study just because of the benefits, but again you have to understand people cannot participate in the study if there are no benefits” (R1/male/RSV/IDI01).

One researcher in the RSV-study, who was also a member of the community engagement advisory team (CAST) for the study, strongly supported provision of more benefits to participants and the broader community. This researcher was the link between the FWs and the study PIs. She accompanied FWs to the field almost every day, and supported the clinical team during home visits. She was particularly frustrated by institutional limitations of what to offer the community, especially when the advice appeared misaligned with livelihood struggles for most households at a time of drought and famine. The field team estimated that nearly half of the community required some form of food aid over the study period, and requested to provide food items to participating households as part of study compensation for the considerable time taken in follow-up visits. After reviewing the situation, the CAST group advised against the request on the grounds that it was not within the overall mandate and focus of the research centre, and because of concerns of intra-community inequity if non-participating households were not also given food rations. Other arguments were that giving food rations in that context might unduly influence participants to join or stay in the study without a good understanding of the research, that future smaller studies could not provide similar levels of benefits, and that other current research in neighbouring areas may face similar demands that cannot be met at that time due to budgetary limitations. The researcher sounded particularly frustrated with these arguments:

“…we have tried to forward these (requests for additional benefits) to the CAST team and tried to justify every small thing we give, but every other time we do so in our CAST meeting, we are told again we can’t (give more benefits). You know, you go there wishing you could be allowed to take a packet of flour to the household, but again our hands are tied” (R2/female/RSV/IDI05).

### Fieldworkers and frontline researchers as gatekeepers to benefits for community members

Based on study protocols and job descriptions, FWs were not expected to negotiate research benefits with participants. However, FWs were the immediate link between the participants and the researchers through which information and requests were communicated. In this intermediary role, FWs seemed to mediate the type and nature of information that was passed on, choosing how information was framed and what was emphasised. As FWs said, they were the ones who often delivered study benefits and tokens of appreciation to participants; they attended to participants at the health facility, helping them fill the various forms, manoeuvre through bureaucracies and ensure access to quick services. It was therefore inevitable that participants would perceive FWs as conduits for study benefits; the people they needed to talk to and negotiate with first. This was described in an FGD with FWs and repeated in other interviews:

FW5: You are the person who goes there handing over those things (benefits) to them. (*FW3 interrupts)*

FW3: And you give the information yourself and whenever they come here, when they want to get the benefit, it’s you they meet and it’s like whenever they meet you everything goes smoothly.

FW4: When they see us, it’s like they have met the benefits!

(FW/RSV/FGD07)

For some FWs, working for the research centre was a means by which they could help improve the livelihood of the community. During DK’s field observation, she reported that most FWs strongly felt that assisting participants and the community gain benefits, especially health care for the really poor households, was one way of helping those households; they seemed to view their role as helping these families alleviate some of their health care burdens. Convincing the researchers to provide additional benefits and to respond to other needs of these participants and the community was one way in which the Research Centre, and hence also the fieldworkers, could be seen to be valuable to the community. Given the daily livelihood struggles that fieldworkers encountered among some participants, the main issue for fieldworkers was not about undue inducement (which seemed abstract) but about responding to these needs.

While FWs were delighted when their requests to increase benefits for participants were accepted, they were also aware of intra-household and intra-community jealousies that could stem from unequal distribution of such benefits. Sometimes these tensions seemed to work in the FWs’ favour particularly where some ‘difficult’ participants later regretted refusing the study. This strengthened, at least temporarily, participants and FW status vis-a-vis non-participants. FWs were also concerned that complex impact of studies on social relations within households and communities might continue and shift after the study ends:

We don’t know what will happen (when the study ends), but what I think is that their relationship [participants and non-participants] at first may not be good, because if somebody had gotten used to being attended to quickly when they get here [dispensary], so their colleagues used to be jealous; now this time when they come and they all queue, there may be some exchange of words … (FW4/Male/RSV/FGD06)

## Discussion

This paper highlights the complexities arising in negotiating for research participation, particularly between fieldworkers and participants, and the centrality of benefits in those negotiations. We have presented the voices and experiences of those who are most affected by policies and decisions of ERCs but who are rarely consulted in benefit discussions; the fieldworkers, research participants and researchers at the frontline of research implementation. There are some limitations to point out. Firstly, the study was not designed to explore benefits; rather the issue of benefits emerged as a theme in a broader social science study that was looking at the nature of interactions between fieldworkers and participants in two community-based studies. Secondly, and relatedly, we are hesitant to make normative claims based on this relatively small study. Nevertheless this paper makes a contribution to wider debates on how benefits are negotiated on the ground, and is feeding into on-going in-depth consultations on benefits in Kilifi that will inform local policy. This paper also highlights the issues - many unaddressed in current guidelines - that staff at the frontline of research implementation face in relation to benefits, particularly when carrying out research in socio-economically disadvantaged populations. It is these issues that the following sections address.

### Study benefits facilitating participant retention in research

As has been widely documented in this setting and elsewhere, study benefits, and especially health care, appeared to greatly influence participants’ decisions to join and stay in the study [26–29]. This is unsurprising in our setting given that many households are quite far from public health care facilities, and that many of these facilities are understaffed, face drug stock-outs, and impose charges [30, 31]. Therefore what might appear to be relatively small benefits for facilities or households (the RSV-study), or indeed large ones (the Malaria-study) can have a significant positive impact on households, such as better care, reduced costs and more accessible health care services. Even small provisions such as sweets and refunds of transport costs are described as benefits and apparently contribute to decision-making surrounding study participation. A challenge is that the information about a study, including risks and inconveniences, may ‘crowd out’ [32] information about, discussions and negotiations over benefits; participants may focus on information about benefits rather than other study information if benefits are their immediate priority. Where benefits are increased over the course of a study in response to requests, individuals may also join future studies in the same area with the expectation that benefits can be negotiated and increased. In both of these scenarios, consent processes may be undermined and – where participants agree to procedures they would otherwise have rejected – there could be concerns of undue inducement [19]. Community engagement is one approach to clarifying the role and different components of research, and how it differs from routine health care. However it may be an inadequate response in a context of such poverty. Employing FWs from the community assists with having a group who can relate to communities at a personal level, and their long term presence in the community can contribute to greater mutual understanding, including regarding what benefits are and why they are provided in research. However this is clearly an ethically and emotionally demanding position for FWs, as discussed more below.

### Types and level of research benefits: a continuing challenge for research in developing countries

It became apparent in this study that the appropriate types and levels of benefits, and to whom such benefits should be provided, were key challenges facing frontline research staff. In particularly, it was not clear how to respond to requests for humanitarian assistance when these were unanticipated. While there were no easy responses to these issues, some researchers preferred adhering to ‘rules’ as outlined in their approved study protocols, whilst others felt some flexibility should be allowed in practice. In certain situations, concerns of undue inducement were perceived to be too narrow a concern when faced with poverty and famine.

These local level debates echo elements of international benefit sharing and ancillary care debates [10, 33], and of recent debates among researchers at the Programme [22]. Some argue that research and research participation should primarily be based on goodwill, altruism, and partnership, and aim to avoid costs to participants and a commercial relationship [13, 34, 35]. Others argue that research should maximise participants’ benefits given the relative wealth of the research institution and multiple community needs [10, 36–38]. As evidenced in this research, there are no easy answers to these issues. Based on the findings of this research, and the literature on the role of the duration and intensity of relationships between researchers and study participants being important in influencing researcher’s responsibilities [10, 39], there are strong ethical arguments that a long term relatively wealthy research centre hosted in a poor community should respond to background inequities and humanitarian emergencies that exacerbate vulnerability of these populations. This could be done through programme-wide and study specific strategies. Working closely with relevant government agencies would be one way of responding to these background inequities, as is currently happening at the research programme, and as described earlier in this paper in relation to the Ministry of Health. However a particular challenge for many researchers including in our setting is how to respond to unmet needs not related to health, where lack of relevant expertise features alongside other concerns related to establishing boundaries around these responsibilities [40], a point we revisit later in this discussion.

At the KEMRI-WT programme, an approach that has informally evolved to respond to background inequities has been to supplement individual benefits with the provision primarily of *medical* benefits to whole communities. This is primarily through collaborations with the Ministry of Health to support sustainability, as well as individual benefits to participants [41]. This compromise, it is hoped, minimises risks of undue inducement for individual participants, protects community harmony, avoids a commercial relationship with participants, and protects and strengthens a key relationship of health researchers with the MOH. This approach is potentially one way to tackle micro-level justice issues [10] in a way that recognises macro-level justice concerns. It is recognised however that this could only ever be one of a set of approaches to benefit sharing at both the micro- and macro-levels. On-going research at the research centre aims to feed into these discourses. Findings from this study support consideration of both community and participant benefits, and of health and non-health benefits especially in cases of emergency humanitarian need. To mobilize resources to support this, one approach proposed by Ballantyne et al. is to levy different infrastructural charge rates for industry and non-industry funded research [13], a proposal that could be explored.

### Implications for fieldworker roles

In this study, it became apparent that fieldworkers were ‘agitating’ for increased benefits to participants partly out of empathy for the livelihood needs of some of the participants, out of good ‘neighbourliness’ as part of social support, and to advance their self-interests (for example, advance their status in the community, or meet recruitment targets). Fieldworkers appearing to overly encourage research participation through emphasis on study benefits can also be seen as responding to real needs of their own communities, in contexts of unmet health needs and underperforming health care systems, as has also been described previously in this setting and elsewhere [12, 16, 42–44]. The challenge is where such support is felt as pressure by participants to join or remain in research, or as the fieldworker - rather than the participant - effectively making the decision to participate in research. FWs’ approach to responding to participants’ (and community) needs could also be perceived as indicating conflicting motivations; to meet recruitment quotas versus to respond to humanitarian situations they encountered. FWs may not have always perceived these motivations as potentially ethically conflicting, but rather as complementary in practice: encouraging participation both improved their study success rate in terms of recruitment rates, and ensured that participants could access health care services and other study benefits. FW sometimes described this as an ‘honourable’ thing to do as they ‘brought development’ to their communities, but it also appeared to strengthen the FWs’ status in the community. It could also be that some FWs were under-supported to unpack and carefully consider the range of potential ethical dilemmas that could arise in their everyday interactions with participants, and possible responses. This strengthens the call for appropriate support to FW and frontline research staff, as discussed below. Such support would be responsive to the context in which FWs work, and could be systematized across the research institution. It could, for example, include skill-building and knowledge-based support (such as training on communication skills, basic research ethics principles), interactive sessions that draw on FWs field experiences to identify and unpack ethical challenges and dilemmas, and more broadly changing the culture of team interactions to support open discussions of issues that could otherwise be perceived as individual failures at work. These and other initiatives could be considered as contributing towards developing ethical mindfulness among research staff [43], including a critical awareness at all levels of their multiple roles and identities. Perceptions of researcher and fieldworker roles and identities may not be shared with community members, and this might contribute to ethically challenging moments in research work.

One recent suggestion from the research centre to facilitate flexibility on the ground and to provide a response system for FWs facing unexpected pressing ethical concerns is to adopt a ‘tsunami fund’ or some form of allowance for FWs, with guidelines on its use [22]. As the authors note, this has its own potential to raise new problems including sourcing and accounting for such funds, establishing fair and equitable systems for FWs to distribute such benefits, possible unexpected or unintended impacts on power relations between FWs and participants, and reducing households’ ability to refuse or withdraw from the study. Moreover, such short-term strategies might leave families worse-off post-study by displacing some other forms of support that families draw upon. Another strategy, potentially a more acceptable approach, is to link such support to other institutions with remit to provide similar assistance such as NGOs, government departments, and CBOs operating in the area. A challenge, however, is the ability and sustainability of those institutions. The dilemma therefore remains: how far should researchers go in addressing real needs of community (beyond health-related needs) and how should they do this in ways that do not leave the community in precarious situations at the end of such support?

### The importance of advisory systems to studies during research implementation

For approved research protocols which allow flexibility on the ground, a continuing challenge for many researchers is guidance on which other types of benefits, above those directly related to the research, to provide. One approach seen as valuable at the research centre is to establish advisory teams for studies (CAST) to guide on unanticipated ethical issues during research implementation. Guidelines on the role and mandate of CAST were formulated, to also safeguard against potential to usurp ERC power and authority. Thus, the CAST supplements institutional and national ERCs in advising on levels and types of benefits being offered by individual studies, given that ERCs at national level are often unable to take into account local day-to-day issues and concerns. By sticking to guidelines and study protocols, some of the CAST members were sometimes perceived to be unresponsive to ground realities, even though CAST are only supposed to advice, with the final decision remaining with the PI. On the other hand by being too flexible, there were concerns that the CAST would lose the objectivity necessary to offer appropriate advice. We found that the CAST played a critical role in providing this objective view, but also that they needed to allow some reasoned flexibility. One approach would be for CAST members independent of the study team to accompany FWs in field visits, and make independent assessments of field situations. While it is important to involve FWs as one group to consult with, it places too large a burden on them in contexts where they are intensely emotionally involved in individual and household needs. their insider knowledge, however, is valuable in informing the decisions that may be taken. The roles of ERC remain crucial in advising on the actions being considered. The ancillary care framework could provide some guidance, where flexibility is possible, on the type and range of benefits as it provides an argument for why researchers might have a responsibility to offer more support in this type of study and context, especially where unpredicted challenges are faced [33]. Having a responsive CAST group or equivalent is one approach to support benefit negotiations at the local level to supplement national approaches and accommodate flexibility on the ground. Such groups would then need careful understanding of when decisions need to be checked with national bodies.

## Conclusion

The issues that this paper raises may be similar in other settings, where research is conducted by relatively wealthy research institutions among low income populations. Debates about researchers and research organizations’ responsibilities in extreme situations of poverty and unmet health needs among host communities increasingly filter into debates on ethical research conduct in developing countries. However the voices of those who find themselves - inevitably because of their interface position - negotiating for research participation, and who often face these situations upfront, the fieldworkers and frontline research staff, are rarely included in such debates. This paper describes the morally imbued work of such staff, and the ethical dilemmas they face working in developing country settings. Beyond providing appropriate support for such staff to be able to respond in these circumstances, we suggest the need for more specific targeted empirical work around how well the current guidelines and frameworks on research benefits are working in developing country settings; and empirically informed arguments for researchers and research institutions’ responsibilities and the limits of these. Current on-going community consultation work at the research centre, with range of stakeholders including community members and fieldworkers, is aimed at providing some guidance on this area.

## Endnotes

^a^As of November 2011.

^b^The SFWs in the two case studies were not direct supervisors of FWs, but had roles that required more experience such as counterchecking quality of samples collected and delivering samples from field to main laboratory offices.

^c^While the Malaria-study had about 400 participants by the time of this research, we excluded about 220 households from the list of potential participants for this research as they were involved in another social science study which some questions similar to those asked in this study. We selected the 30 households for observation based on diversity of locality, of FWs allocated to the households and rota of field visits for FWs.

^d^As indicated in Table 1, the case studies had other researchers apart from the PI. Coordinators and managers are included in this study as ‘researchers’ because they were involved in developing and implementing the research protocol.

^e^This was informed by our experiences of natural group discussions in the RSV-study, in which we noticed that female household members tended to talk less, would often verbally agree with views of the male-members, while sometimes non-verbally seeming to disagree.

^f^The figure is cumulative of 75% in the 5-17 months age-group and 95% in the 6–12 weeks age-group of participants in the Malaria-study who were participating at the time of conducting this social science study.

^g^Prenatal and antenatal services are ideally provided free at the public health facilities; however mothers attending these services often queue for long. They also pay referral costs in case of complications.

## Abbreviations

CAST: Community Engagement Advisory Team
CBO: Community-Based Organization
CE: Community Engagement
CLG: Community Liaison Group
ERC: Ethics Review Committee
FGD: Focus Group Discussions
FW: Fieldworker
IDI: In-Depth Interviews
KHDSS: Kilifi Health Demographic Surveillance System
KWTRP/KEMRI-WT: KEMRI-Wellcome Trust Research Programme
MOH: Ministry of Health
n: number
NFS: Nasopharyngeal Flocked Swabs
NGO: Non-Governmental Organization
P: participants
R: Researcher
RSV: Respiratory Syncytial Virus
SFW: Senior Fieldworker.
